# Comment on: anesthetic management of a pediatric patient with Freeman-Sheldon syndrome undergoing atrial septal defect closure: a case report

**DOI:** 10.1186/s40981-023-00668-y

**Published:** 2024-01-23

**Authors:** Mikaela I. Poling, Craig R. Dufresne

**Affiliations:** 8501 Arlington BLVD, Suite 420, Fairfax, VA 22031 USA

## To the Editor

We read with interest a recently published case of general anesthetic management of an 8-year-old girl who was stated to have Freeman-Sheldon syndrome and underwent atrial septal defect closure [1]. While the authors are to be applauded for their excellent anesthetic management of a patient with a complex presentation, the clinical implications of inaccuracies about the syndrome contained in their report are concerning.

While known by many names through the years, Freeman-Sheldon syndrome is now known as Freeman-Burian syndrome (FBS) to avoid confusion with the similar-appearing Sheldon-Hall syndrome (SHS) [2]. For many years, FBS and SHS were considered a single entity and part of the limb deformity group of disorders known as distal arthrogryposes (DA) [2–4]. FBS, which preferentially affects craniofacial development, is now known to be distinct from SHS, which affects the extremities and spine preferentially [2–5]. Although both are muscle disorders, FBS is considered a complex craniofacial myopathy, with SHS considered a DA [3]. While FBS shows significant variability in severity, it is a single syndrome and neither a group of conditions nor a disease.

As suggested by the literature, FBS has a 30–60% false-positive diagnosis rate [4, 5]. On closer review, it is not surprising the patient appeared to have anatomic hallmarks of SHS and a history supporting this diagnosis over FBS [1]. Facial photographs show a triangular face and a small mouth, and the history includes ease of laryngoscopically introduced endotracheal intubation [1, 4]. Patients with FBS have elongated faces and true microstomia that prevents the use of a laryngoscope [4]. Unfortunately, the authors do not clearly specify the diagnostic criteria (microstomia, pursed lips, deep nasolabial folds, and H or V-shaped chin defect and two major arthrogryposes—typically, camptodactyly with ulnar deviation and equinovarus) [1, 4].

Patients with FBS do not have an elevated risk for malignant hyperthermia or neuroleptic malignant syndrome, the hypothesis for which arose from one report of two cases and another reporting a single patient [6–8]. In FBS, hyperpyrexia may occur in any physiologically stressful situation but responds to ibuprofen [8]. The authors do discuss several relevant aspects of anesthesia care in patients with FBS, such as avoiding respiratory depressing agents and difficult vascular access and airway management [1]. Up-to-date anesthesia care recommendations are available online through OrphanAnesthesia (orphananesthesia.eu) for FBS, SHS, and DA types 1 and 3 [8–11].

Much of the older information concerning FBS is not accurate and continues to be cited in the literature and relied upon in clinical practice. To advance the care for these very complex patients, caution must be taken when selecting articles. We have reviewed twelve case reports published since 2020 that share a similar set of problems easily prevented by searching recent literature.

## Data Availability

Not applicable.

## Abbreviations

DA: Distal arthrogryposis
FBS: Freeman-Burian syndrome
FSS: Freeman-Sheldon syndrome
SHS: Sheldon-Hall syndrome
